# Effects of body weight and alcohol consumption on insulin sensitivity

**DOI:** 10.1186/1475-2891-9-14

**Published:** 2010-03-22

**Authors:** Qiwei X Paulson, Jina Hong, Valerie B Holcomb, Nomeli P Nunez

**Affiliations:** 1 Department of Nutritional Sciences, College of Natural Sciences, the University of Texas at Austin, Austin, Texas, USA

## Abstract

**Background:**

Obesity is a risk factor for the development of insulin resistance, which can eventually lead to type-2 diabetes. Alcohol consumption is a protective factor against insulin resistance, and thus protects against the development of type-2 diabetes. The mechanism by which alcohol protects against the development of type-2 diabetes is not well known. To determine the mechanism by which alcohol improves insulin sensitivity, we fed water or alcohol to lean, control, and obese mice. The aim of this study was to determine whether alcohol consumption and body weights affect overlapping metabolic pathways and to identify specific target genes that are regulated in these pathways.

**Method:**

Adipose tissue dysfunction has been associated with the development of type-2 diabetes. We assessed possible gene expression alterations in epididymal white adipose tissue (WAT). We obtained WAT from mice fed a calorie restricted (CR), low fat (LF Control) or high fat (HF) diets and either water or 20% ethanol in the drinking water. We screened the expression of genes related to the regulation of energy homeostasis and insulin regulation using a gene array composed of 384 genes.

**Results:**

Obesity induced insulin resistance and calorie restriction and alcohol improved insulin sensitivity. The insulin resistance in obese mice was associated with the increased expression of inflammatory markers Cd68, Il-6 and Il-1α; in contrast, most of these genes were down-regulated in CR mice. Anti-inflammatory factors such as Il-10 and adrenergic beta receptor kinase 1 (Adrbk1) were decreased in obese mice and increased by CR and alcohol. Also, we report a direct correlation between body weight and the expression of the following genes: Kcnj11 (potassium inwardly-rectifying channel, subfamily J, member 11), Lpin2 (lipin2), and Dusp9 (dual-specificity MAP kinase phosphatase 9).

**Conclusion:**

We show that alcohol consumption increased insulin sensitivity. Additionally, alterations in insulin sensitivity related with obesity were coupled with alterations in inflammatory genes. We provide evidence that alcohol may improve insulin sensitivity by up-regulating anti-inflammatory genes. Moreover, we have indentified potential gene targets in energy metabolic pathways and signal transducers that may contribute to obesity-related insulin resistance as well as calorie restriction and alcohol-induced insulin sensitivity.

## Background

Obesity and associated metabolic pathologies are the most common risk factors for a variety of diseases leading to mortality, including type-2 diabetes [1,2], hypertension, cardiovascular disease [3,4], and cancer [5]. Obesity is also a risk factor for diseases that cause serious morbidity such as osteoarthritis and sleep apnea [6,7]. Moreover, obese individuals who consume generous amounts of alcohol may be prone to the development of metabolic syndrome, since alcohol can contribute approximately 6% to 10% of the total calories consumed. However, epidemiological data show that moderate alcohol use has several beneficial effects, which include decreasing the risk of type-2 diabetes [2,8]. Alcohol consumption may decrease the risk of type-2 diabetes by promoting insulin sensitivity [9]. It remains to be determined if alcohol consumption improves insulin sensitivity for all body weight phenotypes (e.g., lean, overweight, or obese). Thus, it is not clear if the effects of alcohol consumption on insulin sensitivity are modified by body weight or body fat levels.

Excess calorie consumption can lead to adipocyte hypertrophy and hyperplasia which in turn increases the production and secretion of adipokines (e.g., leptin, adiponectin) and inflammation mediators (e.g., Interleukin 6) by the fat cells [10]. Adipokines and inflammation factors play key roles in the development of insulin resistance [10]. Currently, there are several theories to explain how obesity causes insulin resistance. One theory is that in obesity, the excess adipose tissue produces high levels of adipokines which promote insulin resistance, on the other hand, the expression of factors that promote insulin sensitivity, such as adiponectin are decreased [11,12]. Adiponectin, secreted mainly by adipose tissue, is closely associated with visceral fat rather than subcutaneous fat, and is found at lower concentrations in conditions often associated with insulin resistance such as type-2 diabetes, cardiovascular disease, and metabolic syndrome [13]. The promotion of insulin sensitivity by alcohol consumption has also been associated with an increase in systemic adiponectin levels [12].

Insulin resistance is also shown to be associated with the accumulation of macrophages in white adipose tissue of obese individuals [14]. Factors secreted by fat cells, such as MCP1, initiate the migration of monocytes to white adipose tissue where they develop into activated macrophages [15]. These activated macrophages secrete factors that induce a persistent inflammatory response. The excess production of adipokines and inflammation factors by the adipocytes and macrophages can lead to the development of insulin resistance in key tissues that regulate glucose metabolism, such as skeletal muscle and adipose tissue [16,17]. A second proposed hypothesis suggests that excess fat leads to the accumulation of lipids in non-adipose tissue such as liver and muscle, causing lipotoxicity and eventual insulin resistance [18]. A third hypothesis proposes obesity-dependent insulin resistance occurs through the increased production of reactive oxygen species (ROS) [19]. ROS themselves can induce insulin resistance or adversely affect the production of insulin by the pancreas [20,21].

To better understand the relationship between body fat and insulin resistance, we measured the expression of genes associated with energy homeostasis and insulin regulation using a gene array containing 384 genes linked to obesity and insulin resistance. To this end, we obtained white adipose tissue from mice fed a calorie restricted diet (CR), a low fat diet (LF Control), or a high fat diet (HF), consuming either water or 20% ethanol in the drinking water. Previously, we showed CR, LF Control, and HF diets induced similar body fat phenotypes in mice to those found in individuals considered lean (BMI less than 25), overweight (BMI between 25 and 30), and obese (BMI higher than 30), respectively [22]. Therefore, the present study design allowed us to determine the effects of alcohol consumption on genes linked to obesity and insulin resistance on lean (CR), Control (LF) and obese (HF) mice. We show that obesity induces insulin resistance, while both calorie restriction and alcohol consumption promote insulin sensitivity. Furthermore, calorie restriction and obesity have drastic opposing effects on pro- and anti-inflammatory genes as well as genes involved in adipose tissue energy metabolism such as Lpin2, Kcnj11 and Rbp4. Moreover, alcohol reduces the expression of a different spectrum of inflammatory factors than those regulated by body weight and it significantly upregulated the expression of anti-inflammatory genes. Our current study contributes to a better understanding of how diet modification and alcohol consumption affect insulin sensitivity.

## Materials and methods

### Study Design

Male C57BL/6 mice (The Jackson Laboratory, Bar Harbor, Maine) were housed according to NIH guidelines (National Research Council, 1996) in the Animal Resources Center at the University of Texas at Austin (UT). Animal care was provided in accordance with the procedures outlined in "Guide for the Care and Use of Laboratory Animals" (NIH Publication No. 86-23, 1985). All animal procedures were approved by UT's Institutional Animal Care and Use Committee. Lean, normal, and obese body weights were generated by manipulating caloric intake. At 6 week of age, mice were randomized to receive the following diet treatments for 20 weeks: 1) 30% calorie restricted (CR) regimen (27% protein, 54% carbohydrate, 6% fat; Research Diets, Inc. #D12492) to generate lean mice, 2) low fat diet regimen (19% protein, 67% carbohydrate, 4% fat; Research Diets, Inc. #D03020702) to generate LF normal mice, and 3) high fat diet regimen (26% protein, 26% carbohydrates, 35% fat; Research Diets Inc. #D12450B) to generate obese mice. All mice consumed either water or 20% w/v alcohol in the drinking water throughout the study. Each group was composed of 15 mice.

### StellARray

Total RNA was extracted from WAT from six randomly chosen mice from each group (see qPCR method for details). Quantitative PCR data was collected using the Mouse Diabesity 384 StellARray™ qPCR Array from Bar Harbor Biotechnology (Lonza Cat. number 00188200). GeneSieve System of BHB (Bar Harbor Biotechnology, Trenton, ME) was used for selection of gene candidates included in the array analysis. Briefly, genes were selected by relevance to a biological process. First, databases containing gene-centric biological information were merged. According to this information, over 16 million abstracts were searched for genes involved in a related process. Then genes were ranked by multiple fields, such as quantity and quality of literature references. This was followed by filtering or "sieving" thousands of genes using, for example, canonical signaling pathways, microarray data, gene ontology data, downstream targets of transcription factors, etc. Finally, a list of the most pertinent genes was created and included in the array. Six biological replicates were randomly chosen from each diet group, and total RNA was extracted from epididymal fat tissue. Each StellARray qPCR Array well was loaded with 10 microliters of sample-specific, SYBR Green master mix containing a chemically modified hot-start Taq polymerase (Applied Biosystems, Inc.). The array was heat-sealed and run on a 7900HT Sequence Detection System (Applied Biosystems, Inc.) using default cycling parameters for 40 cycles (1 cycle of 50°C for 2 minutes, 1 cycle of 95°C for 10 minutes, 40 cycles of 95°C for 15 seconds, and 60°C for 1 minute). Fluorescence data was acquired during the 60°C anneal/extension plateau. Post-run data collection involved the setting of a common threshold across all arrays within an experiment, exportation and collation of the Ct values, and analysis via Global Pattern Recognition™ (GPR) software. GPR was designed to take advantage of biological replicates to extract significant changes in gene expression, thus providing a novel alternative to compare the change of expression of a gene normalized to every other gene in the array. By comparing the expression of each gene to every other gene in the array, a global pattern was established, and significant changes were identified and ranked [23].

### StellARray Data Validation by qPCR

The expression of leptin and adiponectin were examined by qRT-PCR. Immediately after removal from the animal, white adipose tissue (WAT) was snap frozen and stored in liquid nitrogen. Total RNA was extracted using RNeasy Lipid Tissue Mini Kit (Qiagen, Hilden, Germany) according to the manufacturer's instructions. The amount and quality of RNA was verified by measuring the absorbance at 260 and 280 nm. Reverse transcription of RNA was performed with the High Capacity cDNA Reverse Transcription Kit (Applied Biosystems, Foster City, CA). Primer pairs were designed using Primer 3 software [24]: Leptin forward: 5'-TCTCCGAGACCTCCTCCATCT-3' and reverse: 5'-TTCCAGGACGCCATCCAG-3'; Adiponectin forward: 5'-AAGGACAAGGCCGTTCTCT-3' and reverse: 5'AGAGTCGTTGACGTTA TCTGCATA-3'. Real-time PCR was performed with a SYBR GreenER qPCR kit (Invitrogen, Carlsbad, CA) and a Mastercycler ep Realplex real-time PCR thermocycler (Eppendorf, Hamburg, Germany). The relative expression level of target genes was normalized to the endogenous reference gene 18 s rRNA. Amplification specificity was confirmed by melting curve analysis.

### Serum leptin levels

Leptin levels were determined with a Luminex-based bead array method using a LINCOplex simultaneous multi-analyte detection system (Linco Research Inc., St. Charles, MO) according to the manufacturer's instructions.

### Body fat levels

Percent body fat levels were measured with dual energy x-ray absorptiometry (DXA) using a GE Lunar Piximus II densitometer (Madison, WI).

### Statistics

For glucose and insulin tolerance tests, statistical analysis of individual diet curves within water and alcohol treatment was performed with Student's unpaired t tests of areas-under-the-curve. Statistical comparisons between water and alcohol groups and for qPCR results were performed using two-way ANOVA. A p value of <0.05 was considered statistically significant. All statistical analyses were performed with SPSS software (Chicago, IL). Array data analysis and statistical significance were calculated using the Global Pattern Recognition™ (GPR) software mentioned earlier.

## Results

### Establishment of different body weights

**Figure 1:**
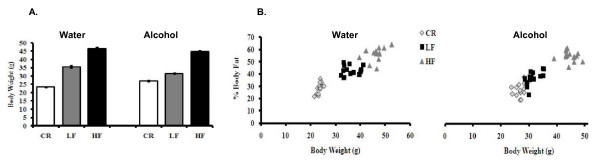
**Body weight and percent body fat in mice consuming water and alcohol**. **Panel A**: Body weight in animals consuming the CR, LF, and HF diets and drinking water or 20% alcohol. **Panel B**: Percent body fat levels in the various groups of mice.

After 12 weeks on the diet regimen of 30% calorie restriction (CR), low fat (LF) or 35% high fat (HF) diets, CR mice had the lowest body weight, LF mice had an intermediate body weight, and HF mice the highest body weight (Fig 1A). We also show that body weights correlated directly with body fat levels. Mice with the lowest body weight had the lowest percentage body fat, and mice with the highest body weight had the highest percentage body fat (Fig. 1B).

There were no significant differences in baseline body weight between CR, LF, and HF mice. Moreover, final body weight between water and alcohol consuming CR, LF, and HR mice tended to be similar, suggesting that alcohol consumption did not affect the susceptibility of gaining weight. To explain the lack of difference in body weight after consuming a relatively large amount of alcohol, we measured calories consumed from both food and alcohol for each group of mice. Previously, we showed that alcohol-consuming mice tended to consume fewer calories from food but more total calories (calories from food + calories from alcohol) than water-consuming mice. The alcohol-consuming mice received about 18-35% of the total calories from alcohol [25]. Despite the fact that alcohol-consuming mice consumed more overall calories, their body weight and percentage body fat were not significantly different from those of water-consuming mice [25]. This result indicates that alcohol regulates insulin sensitivity without significantly affecting body weight or body fat level.

### Leptin mRNA levels were affected by body weight and alcohol consumption

**Figure 2:**
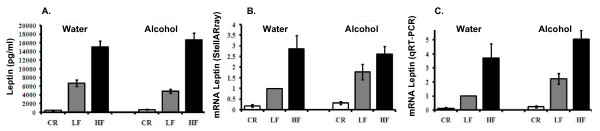
**Leptin mRNA and protein levels**. **Panel A**: Serum leptin levels. **Panel B**: Adipose tissue leptin mRNA levels measured by StellARray. **Panel C**: Adipose tissue leptin mRNA levels measured by qRT-PCR. The data in this Fig is the relative mRNA change in selected genes in the adipose tissue of CR, LF, and HF mice consuming water or 20% alcohol relative to LF mice consuming water. If the ratio is higher than 1, the mRNA content for that gene was amplified; if the ratio is below 1, the mRNA content decreased. The ratio for the LF mice consuming water is 1 because the mRNA level for that gene was the control group.

Both leptin mRNA and protein levels correlated directly with body fat [26]; that is, lean mice had the lowest levels and obese mice had the highest levels (Fig. 2). Results showed that leptin levels increased almost three-fold in the obese mice and decreased 5.5-fold and 3.1-fold, respectively, in water- and alcohol-consuming CR mice, compared to the water-consuming LF (Control) mice; p < 0.05 (Fig. 2A, Table 1). Alcohol consumption increased leptin mRNA levels in adipose tissue approximately two-fold in LF mice. Further qRT-PCR results confirmed leptin levels observed in the array study (Fig. 2B, C). HF mice had the highest and CR mice the lowest leptin expression.

**Table 1:** Genes regulated by body weight/diet and alcohol consumption.

Category/Gene	Change					
	
	Water			Alcohol		
	CR	LF	HF	CR	LF	HF
Metabolism						
Lep	-5.5	1	2.9	-3.1	1.8	2.6
Fbp2	-1.4	1	1.4^a^	-1.6	-1.8	1.5^a^
Gck	2.7	1	-2.9	2.6	2.3	-1.8
Ins1	3.9	1	-2.5	2.8	2.5	-2.7
Pltp	2.4	1	1.0^a^	2.5	1.9	1.2^a^
Apob	4.4	1	-1.7	3.6	1.6	-1.3^a^
Pck1	-1.5	1	1.4^a^	-1.6	-1.7	1.5
Ppp1r3a	-1.8	1	1.4^a^	-1.5	1.6	1.4
Aldh3a2	11	1	-5.8	9.5	3.1	-3.8
Lpin3	1.1^a^	1	3.4	1.0^a^	1.5	2.6
Lepr	-1.6	1	4	1.1^a^	1.5	2.4
Lpin2	8.3	1	-3.3	7.1	2.2	-2.3
Mlxipl	1.2^a^	1	-1.9	1.1^a^	1.8	-1.6
Apoa4	4.1	1	1.0^a^	4.2	1.7	1.1^a^
Mthfr	-2.5	1	1.1^a^	-2.4	-2.9	-1.2^a^
Akt2	2.1	1	1.1^a^	2	2	1.1^a^
Flot2	2.4	1	-1.3^a^	2.2	2.1	-1.2^a^
Nmu	-1.4^a^	1	3.3	1.2^a^	-1.6	2.7
Adrbk1	5.7	1	-1.3^a^	4	2.5	1.1^a^
Sst	2.9	1	-1.1^a^	2.5	2.5	1.1^a^
Adrb2	7.8	1	1.5	5.9	1.6	1.3^a^
Prl	11	1	-4	9	2.3	-2.4
Npy2r	3.2	1	1.2^a^	2.4	2	1.2^a^
Pro-inflammation						
Cd68	1.0^a^	1	4.3	1.9	1.5	2.8
Ccr2	8.3	1	-1.4^a^	7.1	2.9	-1.4^a^
Anti-inflammation						
Il10	4	1	1.5^a^	4.8	2.7	1.2^a^
Ptgds	3.7	1	1.1^a^	3.5	1.6	1.2^a^
Adrbk1	5.7	1	-1.3^a^	4	2.2	1.1^a^
Signal transducers, Growth factors						
Raf1	1.0^a^	1	2.1	1.0^a^	2.1	2.3
Pde3b	-1.8	1	2.1	-1.8	-2.2	1.7
Pde3a	3.2	1	-1.1^a^	2.9	1.6	-1.2^a^
Gne	2.9	1	-1.1^a^	2.9	2.2	1.1^a^
Ptpn2	2.8	1	1.1^a^	1.8	1.5	-1.1^a^
Cblb	1.6^a^	1	-2.3	2.8^a^	3.4	-1.1^a^
Ptpn6	2.4	1	-1.6	1.8	1.8	-1.4
Ppp1cb	-1.8	1	1.8	-1.8	-1.4	1.6
Dusp9	19.2	1	-1.7	17	3.1	-1.2^a^
Itgb3	2.5	1	1.1^a^	2.4	2.4	1.2^a^
Prkx	5.9	1	2.9	4.3	2.3	1.8
Prkcz	4.3	1	-1.8	4.5	3.2	1.0^a^
Nras	5	1	-1.8	4	2	-1.6
Igfbp3	2.4	1	1.2^a^	2	3.1	1.0^a^
Miscellaneous						
Eif4ebp2	4.5	1	-2.1	2.9	2.2	-1.6
Serpina6	3.5	1	-2.1	1.8	1.6	-1.7
Atp2a3	2.9	1	1.1^a^	3.3	2.5	1.2^a^
Stx1a	2.6	1	-1.6	1.9	1.5	-1.5^a^
Alms1	2.5	1	1.1^a^	2.8	2	1.2^a^
Sct	-2.8	1	1.0^a^	-2.6	-2	1.0^a^
Unc13a	3.6	1	-1.4^a^	2.7	1.5	-1.4^a^
Alb	3.3	1	-4	3.5	2.7	-3.4
Bbs1	1.0^a^	1	2.2	-1.2^a^	-1.7	1.8

^a ^No significant change compared to Control. All others were significantly different compared to control mice, p < 0.05.

**Figure 3:**
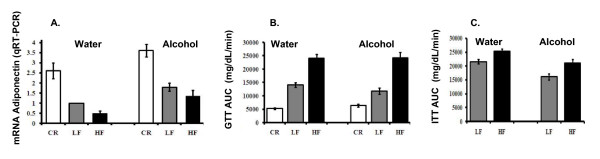
**Adiponectin and markers of insulin sensitivity**. **Panel A**: Adipose tissue adiponectin mRNA levels measured by qRT-PCR. **Panel B**: GTT area under the curve. **Panel C**: ITT area under the curve.

We also measured the effects of body weight and alcohol consumption on adiponectin levels. Adiponectin has been shown to increase insulin sensitivity [27] and its levels are inversely proportional to insulin resistance and type-2 diabetes [28]. Results showed that mRNA adiponectin levels increased 2.5- and 3.5-fold in the white adipose tissue of water and alcohol consuming CR mice, respectively (Fig 3A). In contrast, adiponectin mRNA levels decreased approximately 2-fold in the water-consuming HF mice compared to the Control group (LF mice consuming water). In LF mice consuming alcohol, adiponectin levels were increased by 1.8-fold compared to the Control group (LF mice consuming water; Fig. 3A). In conclusion, alcohol consumption increased adiponectin levels.

### Glucose and insulin tolerance tests

To assess the effects of body weight and alcohol on glucose regulation and insulin sensitivity, we deduced area under the curve (AUC) for GTT and ITT the. AUC is the area between the graph curve and the x-axis between baseline and 120 minute glucose time points. AUC was calculated from GTT and ITT data previously published from our laboratory [25]; however, the AUC data has not been published. Data from the GTT show that mice consuming water had the following glucose AUC (mg/dL/min): 5.3 ± 0.3 × 10^3^, 14.2 ± 0.8 × 10^3^, and 24.2 ± 1.4 × 10^3 ^for CR, LF, and HF, respectively; p < 0.05. Values for the mice consuming alcohol were: 6.4 ± 0.5 × 10^3 ^for CR, 11.8 ± 1.1 × 10^3 ^for LF, and 24.3 ± 2.1 × 10^3 ^for HF; p < 0.05 (Fig. 3B). Results showed that the area under the curve (AUC) for ITT for the water-consuming mice was 21.6 ± 0.8 × 10^3 ^and 25.4 ± 0.8 × 10^3 ^for LF and HF p < 0.05, respectively; and for the alcohol-consuming group was 16.2 ± 1.1 × 10^3 ^for LF and 21 ± 1.4 × 10^3 ^for HF, p < 0.05 (Fig. 3C). Confirming the GTT AUC results, obese mice showed decreased insulin sensitivity in both water- and alcohol-consuming groups. Furthermore, alcohol consumption increased insulin sensitivity in both LF and HF mice, as shown by the lower ITT AUC for both LF and HF mice consuming alcohol. The ITT test was not performed on CR mice because an additional decrease of preexisting low blood glucose levels causes severe hypoglycemia, which can lead to death of the mice (laboratory observation).

### HF and CR mice had opposite inflammatory factor profiles

**Figure 4:**
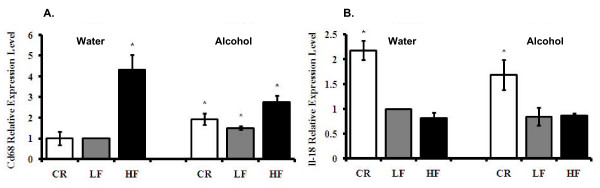
**Adipose tissue inflammation-related factors**. **Panel A**: Il-6 levels. **Panel B**: Il-10 levels. **Panel C**: Il-1α levels. **Panel D**: Adrbk1 levels. The data is fold change in mRNA of gene relative to LF mice consuming water. *Significant difference compared to the water-consuming Control group, p < 0.05.

**Figure 5:**
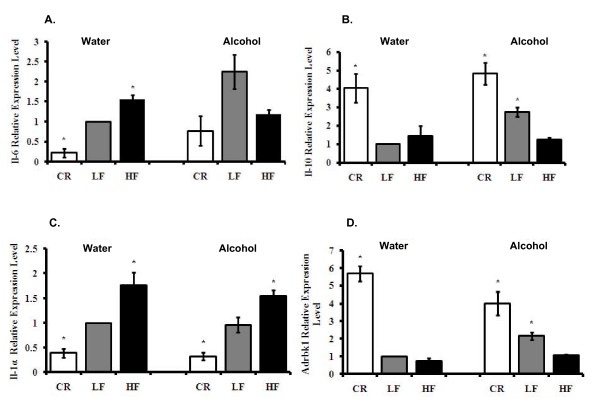
**Adipose tissue Cd68 and Il-18 mRNA expression levels**. **Panel A**: Cd68 levels. **Panel B**: Il-18 levels. The data represent the expression of mRNA relative to LF mice consuming water. *Significant difference compared to the water-consuming Control group, p < 0.05.

Adipose tissue plays important roles in inflammation and is an important initiator of the inflammatory response [29-31]. Currently, one of the most studied phenomena is how obesity-related inflammation affects insulin sensitivity and causes morbidities such as diabetes, cardiovascular disease and cancer. To investigate whether and how alcohol interferes with body weight in regulating obesity-related inflammation, we measured markers of inflammation in mouse WAT. The LF water-consuming group was used as the Control, and therefore the expression level was set to one. The data showed that pro-inflammatory factors including Il-6, Il-1α, and Cd68 were up-regulated in HF mice; and in CR mice, the expression of Il-6 and Il-1α were down-regulated compared to LF Control mice; p < 0.05. CR treatment did not affect Cd68 expression (Table 1, Fig. 4 and 5). Among these inflammatory factors, the expression of Il-6 and Il-1α was not affected by alcohol consumption, while macrophage marker Cd68 expression was slightly up-regulated by alcohol consumption (Fig. 4 and 5, Tables 1 and 2).

**Table 2:** Genes affected by body weight and diet.

Category/Genes	Change			
	
	Water		Alcohol	
	
	CR	HF	CR	HF
Metabolism				
Enpp1	3.1	-1.1^a^	2.2	-1.3^a^
Lrp5	-1.1^a^	4.9	-1.1^a^	2.8
Osbpl1a	-1.4^a^	3.9	-1.3^a^	3.1
Dgat1	1.2^a^	2.1	1.1^a^	1.7
Prkaa1	1.9	1.2^a^	1.6	1.0^a^
Fto	1.1^a^	5.3	1.2^a^	3
Fasn	-1.1^a^	6.4	1.1^a^	3.4
Kcnj11	20.1	-6.9	11.7	-5.3
Gys	-2.6	3.6	-1.5^a^	3.6
Pfkm	4	-1.9	2.9	-1.5^a^
Ucp3	1.9	1.0^a^	1.7	1.0^a^
Cyp19a1	5.6	1.8	3.4	1.5^a^
Cp	1.0^a^	2.6	1.4^a^	1.7^a^
Rbp4	-6.3	1.8	-2.7	1.8
Osbp	4.7	-1.3^a^	2.7	-1.4^a^
Angptl6	2.2	-2.2	1.0^a^	-2.7
Gal	1.2^a^	2.2	1.3^a^	1.5^a^
Ghr	-1.3^a^	4.5	1.0^a^	3.9
Adm	1.9	1.1^a^	1.8	1.0^a^
Foxo1	1.4^a^	1.9	1.9^a^	2.2
Pro-Inflammation				
Il6	-4.5	1.6	-1.3^a^	1.2^a^
Il1a	-2.6	1.8	-3.1	1.6
Il18	2.2	-1.2^a^	1.7^a^	-1.1^a^
Sele	-1.6	1.2^a^	-1.8	1.1^a^
Tlr2	1.8	-1.5^a^	1.6^a^	-1.3^a^
Signal Transducers, Growth factors				
Ptpra	-1.4^a^	14.2	-1.3^a^	9
Ptk2b	1.2^a^	-1.9	1.0^a^	-1.6
Shc2	1.1^a^	1.8	1.0^a^	1.4^a^
Igfbp1	1.2^a^	-1.9	1.0^a^	-1.8
Hras1	1.8	-1.4^a^	1.5^a^	-1.2^a^
Cbl	-1.2^a^	2.1	-1.2^a^	2.4
Src	2.6	1.5^a^	2.1	1.1^a^
Inppl1	3.6	1.2^a^	2.9	1.4^a^
Trib3	2.3	-1.1^a^	1.9	1.0^a^
Pik3ca	1.7	1.2^a^	1.2^a^	1.0^a^
Pik3cb	3.1	1.1^a^	2.5	1.0^a^
Sos1	2.1	1.2^a^	1.9	-1.2^a^
Pik3r2	2.9	-1.4	2	-1.4
Mchr1	-2.2	5	-1.8	4.1
Sdc3	-1.2^a^	3.5	-1.2^a^	3.2
Cblc	1.0^a^	2.7	-1.3^a^	1.9
Miscellaneous				
Foxa1	2.7	-1.2^a^	2.3	-1.2^a^
Trf	3.1	1.4^a^	2.1	1.1^a^
Nat1	2.7	-1.5^a^	2.2	-1.3^a^
Cav3	2.2	1.1^a^	1.8	1.0^a^
Eif4ebp1	-1.3^a^	-1.4^a^	-2.1	1.3^a^

^a ^No significant change compared to Control. All others were significantly different compared to control mice, p < 0.05.

The expression of inflammatory-related factors resistin-like protein β (Retnlb) and macrophage migration inhibitory factor (Mif) were affected by alcohol consumption but not by body weight. The expression of Retnlb and Mif were reduced by alcohol consumption in LF mice; the expression of Mif was also reduced by alcohol consumption in HF animals as shown in Table 3. This indicates that alcohol improves insulin sensitivity by regulating distinct inflammatory pathways independent of body weight.

**Table 3:** Genes affected by alcohol consumption.

Category/Gene		Change		
		**Alcohol**		

		**CR**	**Con**	**HF**

Metabolism				
	Hsd11b1	-1.4^a^	-1.8	1.0^a^
	Apoa1	-1.5^a^	-1.5	-1.5^a^
Pro-inflammation				
	Retnlb	-1.3^a^	-1.5	1.1^a^
	Mif	-1.3^a^	-1.7	-1.4^a^
Signal transducers, Growth factors				
	Prkar1b	1.3	1.9	1.0
Hormone				
	Crh	1.1^a^	-1.5	-1.1^a^
	Ren1	-1.2^a^	-1.7	-1.4^a^
	Gast	-1.3^a^	-1.6	1.0^a^
Miscellaneous				
	Htr2a	-1.5^a^	-2.0	1.3^a^
	Cartpt	2.4	2.7	-1.1^a^
	Npy	1.3^a^	1.8	1.3^a^

No significant change compared to Control. All others were significantly different compared to control mice, p < 0.05.

To further explore the effects of body weight and alcohol consumption on adipose tissue-associated inflammation, we analyzed the expression of genes that have an anti-inflammatory function. Among the anti-inflammatory factors, Il-10 and β-adrenergic receptor kinase 1 (Adrbk1) expression levels were increased in CR animals in both water- and alcohol-consuming groups compared to Control mice; p < 0.05 (Table 1, Fig. 4B and 4D). Increased body weight and body fat had a slight suppressive effect on the expression of these factors, but the changes were not significant. Alcohol consumption increased the expression levels of Il-10 and Adrbk1 in LF mice; p < 0.05 (Table 1, Fig. 4B and 4D).

Taken together, the above results show that body weight regulates a specific spectrum of pro-inflammatory factors including Il-6, Il-1α, and Cd68 and anti-inflammatory factors Il-10 and Adrbk1. CR down-regulated the pro-inflammatory and up-regulated the anti-inflammatory factors; meanwhile, HF up-regulated the pro-inflammatory factors and had a tendency to down-regulate anti-inflammatory factors. Alcohol consumption increased anti-inflammatory factors Il-10 and Adrbk1 but reduced a distinct spectrum of inflammation-related factors including Retnlb and Mif.

### Pro-inflammatory factor Il-18 was increased by CR intervention

Compared to Control mice, we noted that pro-inflammatory factor Il-18 expression had a more than two-fold increase in CR water-consuming mice and a two-fold increase in CR alcohol-consuming mice; p < 0.05. The expression of Il-18 was not affected by alcohol consumption or HF (Table 2, Fig. 5B). In agreement with our findings, earlier studies showed *Il-18*-deficient mice developed obesity, insulin resistance, and hyperglycemia [32]. Our findings, together with previous findings, suggest that Il-18, as a pro-inflammatory factor, does not contribute to HF-induced insulin resistance at the expression level. On the other hand, it is a potential protective factor for insulin sensitivity in CR intervention. Alcohol consumption had no obvious effect on Il-18 expression, further indicating that alcohol consumption and body weight regulation affect insulin sensitivity through distinctive inflammation pathways.

### CR intervention up-regulated prostaglandin D2 synthase (Ptgds)

**Figure 6:**
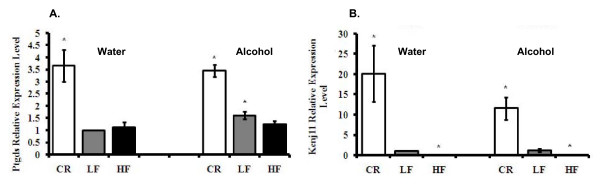
**Panel A: Adipose tissue mRNA Ptgds levels**. **Panel B**: KCNJ11 levels. The data represent the expression of mRNA relative to LF mice consuming water. *Significant difference compared to the water-consuming Control group, p < 0.05.

Lipocalin-type prostaglandin D2 synthase (L-Ptgds) is a glutathione (GSH)-independent PGD synthase that catalyzes the conversion of prostaglandin H2 (PGH2) to PGD2 in the presence of various sulfhydryl compounds. The enzyme is responsible for the biosynthesis of PGD2 in the brain [33]. Ragolia et al. showed that *L-ptgds *knockout mice, among other phenotypes, became glucose intolerant and insulin resistant [34]. L-ptgds is mostly found in the brain tissue; expression of L-type Ptgds and its possible functions in other tissues have not been studied extensively. We showed in this study that CR intervention significantly up-regulated Ptgds mRNA level by more than three-fold in adipose tissue; p < 0.05 (Fig. 6A). Obesity did not affect Ptgds expression in adipose tissue. Alcohol consumption alone increased the mRNA level of Ptgds by 1.6-fold in LF mice; p < 0.05, but alcohol did not have an additive or synergistic effect with different diet regimens on Ptgds expression (Table 1, Fig. 6A). Ptgds was up-regulated by CR and alcohol, indicating the protective roles that adipose tissue Ptgds plays in CR- and alcohol-induced insulin sensitivity.

### KATP channel subunit Kcnj11 mRNA was induced by CR diet and suppressed by HF diet

It has been established that in pancreatic β-cells, KATP channels couple glucose metabolism to insulin release [35]. A number of activating mutations and an E23K polymorphism of *Kcnj11 *are related to the development of insulin resistance and type-2 diabetes [36,37]. Besides pancreatic β-cells, the protein products of *Kcnj11*, Kir6.2, are expressed in the heart, muscle, and brain [38]. To date, no one has shown the relationship between Kcnj11 and adipose tissue. This study showed that Kcnj11 was expressed in adipose tissue, and its expression was affected by body weight. In water-consuming mice, CR intervention increased Kcnj11 mRNA levels by 20-fold, while HF diet decreased its expression by almost seven-fold compared to Control mice; p < 0.05 Fig. 6B. Similarly, in alcohol-consuming groups, CR mice had a more than 11-fold increase, while HF mice had an approximately five-fold decrease in Kcnj11 mRNA expression levels compared to Control mice; p < 0.05 (Table 2, Fig. 6B). Alcohol consumption alone did not change the level of Kcnj11 expression.

### Body weight and alcohol consumption distinctively regulated lipin family member expression

**Figure 7:**
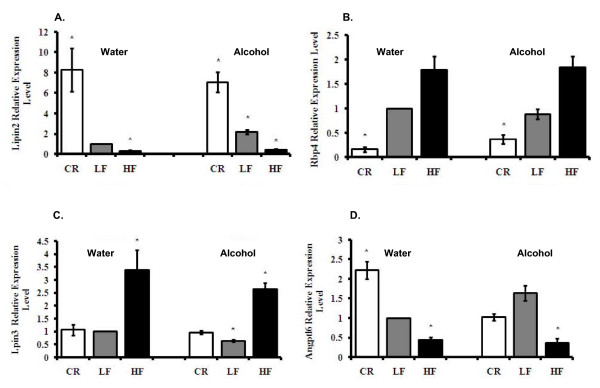
**Hormones and enzymes related to obesity-induced insulin resistance**. **Panel A**: Lpin2 levels. **Panel B**: Rbp4 levels. **Panel C**: Lpin3 levels. **Panel D**: Angptl6 levels. The data represent the expression of mRNA relative to LF mice consuming water. *Significant difference compared to the water-consuming Control group, p < 0.05.

Among genes involved in metabolism and homeostasis, one interesting finding from this study showed that members of the lipin family are distinctively regulated by body weight and alcohol consumption. For mice in water and alcohol groups, Lpin2 expression levels were largely suppressed by HF diet, with a two to three-fold decrease in expression; p < 0.05. In contrast, Lpin2 levels were up-regulated seven-fold to eight-fold by CR intervention; p < 0.05 (Fig. 7A, Table 1). In LF mice, alcohol consumption induced a more than two-fold increase in Lpin2 mRNA levels; p < 0.05. On the contrary, HF mice showed a three-fold increase in Lpin3 expression (p < 0.05), while CR and alcohol consumption had no obvious effect (Fig. 7C, Table 1). Unlike the other two members of the lipin family, white adipose tissue Lpin1 expression was not significantly affected by either body weight or alcohol consumption (data not shown).

### Retinol binding protein-4 (Rbp4) expression in adipose tissue was tightly correlated with body weight

Recent studies have shown a new role for RBP4 as a signal that links adipose tissue dysfunction to systemic insulin resistance and type-2 diabetes [39]. The exact relationship between WAT Rbp4 mRNA level, body weight, and insulin resistance is not clear. Recent studies by others suggest that RBP4 expression is not related to increased body weight; however, RBP4 is strongly related to adipose tissue inflammation [40]. We showed in this study that obesity slightly increased the Rbp4 mRNA in adipose tissue (Fig. 7B). Conversely, there was a significant decrease in Rbp4 mRNA levels in CR mice. In water- and alcohol-consuming CR mice, Rbp4 mRNA was decreased by six-fold and three-fold, respectively; p < 0.05 (Table 2, Fig. 7B). Alcohol consumption did not affect Rbp4 expression levels.

### Angiopoietin-like protein 6 mRNA level negatively correlated with body weight

Angiopoietin-like (Angptl) family of proteins 1-6 are reported to regulate glucose and lipid metabolism and are potential new targets for metabolic syndrome therapy [41]. In adipose tissue, the expression of Angptl3 and 4 were not regulated by different diet regimens and alcohol consumption (data not shown). However, we show that Angptl6 mRNA was induced 2.2-fold in the adipose tissue of CR water-consuming mice compared to Control mice, and suppressed by more than two-fold (p < 0.05) in HF mice in both water- and alcohol-consuming groups (Table 2 and Fig. 7D).

### Dual specificity phosphatase 9 (Dusp-9/Mkp-4) and protein-tyrosine phosphatase, receptor-type, alpha (Ptpra) mRNA level were oppositely regulated by CR and HF diet

**Figure 8:**
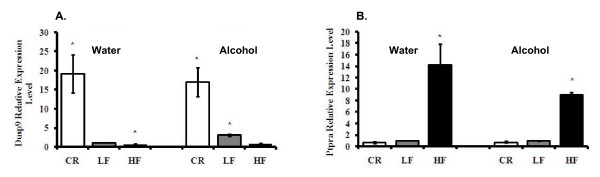
**Expression levels of phosphatases associated with the insulin signaling pathway**. **Panel A**: Levels of Dusp-9/Mkp-4. **Panel B**: Ptpra mRNA levels. The data represent the expression of mRNA relative to LF mice consuming water. *Significant difference compared to the water-consuming Control group, p < 0.05.

Previous studies have shown a protective role for Dusp-9 against insulin resistance through inhibition of stress-activated kinases (SAPs) [42]. Results in Fig. 8A show that Dusp-9 mRNA increased 19-fold and 17-fold in water- and alcohol-consuming CR mice, respectively, compared to Control mice; p < 0.05. In contrast, HF mice had a decreased in Dusp-9 expression by almost two-fold; p < 0.05. Dusp-9 mRNA was increased three-fold by alcohol consumption; p < 0.05 (Table 1, Fig. 8A). Protein tyrosine phosphatase receptor-type alpha (Ptpra) is a phosphatase that inhibits insulin signaling [43]; we show that Ptpra expression was up-regulated by HF diet. In water- and alcohol-consuming HF mice, the expression of Ptpra was increased by 14-fold and nine-fold, respectively; p < 0.05. CR slightly decreased Ptpra expression by over one-fold, although the changes were not significant. Alcohol did not affect the expression of Ptpra (Table 2, Fig. 8B).

## Discussion

We and others have previously shown that obesity induces insulin resistance. In this study, we confirmed this effect of obesity on insulin resistance and provide further evidence that CR and alcohol consumption increase insulin sensitivity. To understand the mechanism behind this phenomenon, we screened the expression of genes related to the regulation of energy homeostasis and insulin regulation in order to determine pathways by which body weight and alcohol consumption affect insulin sensitivity. Our results show that alcohol increased leptin levels in adipose tissue and increased insulin sensitivity in LF mice simultaneously. Leptin is an adipocyte hormone that acts to decrease food intake and increase energy expenditure [44]. Alcohol increased leptin levels without affecting body weight and percent body fat, suggesting that alcohol may increase insulin sensitivity via leptin, which may increase glucose catabolism in alcohol consuming mice. Alternatively, alcohol may increase insulin sensitivity by increasing adiponectin levels, adiponectin has been shown to increase insulin sensitivity [12], and alcohol consuming mice had higher levels of adiponectin (Fig. 3A), suggesting that alcohol consumption may promote insulin sensitivity via adiponectin.

To determine whether insulin sensitivity and glucose homeostasis are modified by alcohol, we performed the GTT and the ITT on mice. The GTT measures how quickly injected glucose is cleared from the blood. Insulin is a hormone secreted by the β cells within the pancreas as a response to blood glucose alterations. Result show that the GTT AUC was not affected by alcohol suggesting that alcohol in the drinking water did not affect insulin secretion by the β cells within the pancreas. To determine if alcohol consumption promoted insulin sensitivity, we performed the ITT on mice drinking water or 20% alcohol. The ITT determines how quickly endogenous glucose is cleared from the blood as a response to insulin administration. Results show that the ITT AUC values were significantly reduced in alcohol consuming mice suggesting that alcohol consumption affects insulin sensitivity by affecting the insulin signaling pathway.

Herein, we selectively discussed genes with the most statistically significant changes and those associated with the insulin signaling pathway, energy homeostasis, and inflammatory response. Other genes affected by body weight or alcohol consumption are listed in Tables 1, 2, and 3 for reference. Unlike most previous studies, we included lean mice in addition to the normal and obese body weights. The significance of including body weight at both ends of the spectrum, lean and obese, is to show not only the deleterious effects of obesity in related metabolic pathways but also the possible protective effects of lean body weight. We show that the CR increased the expression levels of anti-inflammatory genes Il-10 and Adrbk1 (Fig. 3B and 3D) and energy homeostasis-related genes such as Ptgds (Fig. 6A), Lpin2 (Fig. 7A), Angptl6 (Fig. 7D), Kcnj11 (Fig. 6B), and Dusp9 (Fig. 8A) but were not affected by obesity in HF mice. These results provide further insight into pathways with which to study the protective effects of these genes. Another purpose of the study was to investigate the interaction between alcohol and body weight in regulating insulin sensitivity.

We show that in white adipose tissue of obese mice, pro-inflammatory factors including Il-6, Il-1α, and macrophage marker Cd68 (Tables 1 and 2) were elevated. Conversely, CR had an opposite effect on most inflammatory factors such as Il-6 and Il-1α, except for Cd68, which was not affected by CR. This suggested that that HF diet up-regulated macrophages and macrophage-mediated secretion of inflammatory factors Il-6 and Il-1α, while CR protected against inflammation through reduced macrophage activity. We show that alcohol regulated a different profile of pro-inflammatory factors compared to body weight. These factors include Retnlb and Mif. Also, alcohol consumption increased anti-inflammatory factors Il-10 and Adrbk1, which were also increased by CR. This suggested that alcohol and lean body weight might affect the same anti-inflammatory factors to stimulate insulin sensitivity.

Diet intervention may also affect insulin sensitivity by affecting other anti-inflammatory factors such as L-Ptgds. Ragolia et al. showed that *L-Ptgds *knockout mice become glucose intolerant and insulin resistant [34]. Moreover, *H-Ptgds *(Hemopoietic type prostaglandin D2 synthase) null mice display a severe inflammatory response compared to wild type mice [45]. Our results show that L-Ptgds expression in adipose tissue was significantly up-regulated by more than three-fold in CR mice (Fig. 3A). We speculate that increased expression of L-Ptgds played a protective role in insulin sensitivity in CR mice. The anti-inflammatory effects of H-Ptgds are mediated by the cyclopentenone prostaglandins (PGs), including 15-deoxy-Delta(12,14)-PGJ2 (15d-PGJ2), a dehydrated product of PGD2 [42]. Since the diverse effects of L-Ptgds and H-Ptgds result from tissue-specificity, instead of enzymatic function [34,45], we do not eliminate the possibility that in adipose tissue, L-Ptgds triggers an anti-inflammatory effect by producing cyclopentenon PGs. Further studies characterizing Ptgds deletion or over-expression in adipose tissue would be helpful in confirming the relationship between Ptgds expression and inflammation. Besides interfering with inflammatory pathways, such as the NF-κB pathway [45], it is also speculated that L-Ptgds may suppress inflammation by inhibiting monocytes in adipose tissue of CR mice. In fact, Il-6 and Il-1α levels were reduced in CR mice. Interestingly, alcohol increased the level of Ptgds even though it did not have an additive effect on CR alcohol-consuming mice. This further indicated that CR and alcohol affect overlapping factors that regulate inflammation and immune responses.

We further explored genes associated with energy homeostasis and insulin signaling. Lipin family members play important roles in lipid and glucose metabolism, adipocyte development, and inflammation [46]. *Lpin1 *was the first gene identified in this family. Lpin1 mRNA is expressed at high levels in adipose tissue and is induced during differentiation of 3T3-L1 pre-adipocytes [46]. A second member of this family is Lpin2. Little is known about the third member of the lipin family, Lpin3. Here, we found that Lpin1 was not affected by either body weight or alcohol consumption. Interestingly, although they are family members, Lpin2 and Lpin3 had contrasting expression patterns in WAT from CR and HF mice. HF diet suppressed Lpin2 and increased Lpin3 expression. Alcohol increased Lpin2 and decreased Lpin3 expression in the LF mice compared with Control. This suggests opposing roles of Lpin2 and Lpin3 in regulating energy homeostasis and insulin sensitivity. Previous findings show a homozygous mutation of the human *Lpin2 *gene leads to inflammation of the bone and skin [46]. Combined with our findings, Lpin2 may contribute to increased insulin sensitivity by protecting against inflammatory reactions. Based on this finding, further studies on the relations of Lpin2 and Lpin3 expression levels versus insulin sensitivity regulation, as well as inflammatory factor profiles, will help better understand the role the lipin family plays in body weight and alcohol-regulated insulin sensitivity.

Another protein family that adds to the increasingly complex network of hormonal regulation of glucose and lipid homeostasis is the Angptl protein family. Among the six members, Agnptl6 has been shown to promote angiogenesis and wound closure led by keratinocyte proliferation [47]. Angptl6 has also been shown to have metabolic functions. Others have show that loss of Angptl6 in early development is lethal and those mice that survive developed excess adiposity, adipocyte hyperplasia, and increased circulating and tissue deposition of free fatty acids [48]. In support of the above findings, our data showed that in adipose tissue, Angptl6 mRNA was induced by CR intervention and down-regulated by obesity in HF mice. Moreover, previously we showed that CR mice healed inflicted wounds faster and obesity inhibited wound healing in mice, thus suggesting that factors like Angpt16 may promote wound healing in CR mice but in obese mice the decreased Angpt16 may impair wound healing [49]. Similar observations as these have been noticed in humans studies [50].

We investigated signaling pathways related to insulin function. The JNK pathway is one of the signaling pathways linked to insulin signaling [51,52]. JNK kinase can inhibit the signaling pathway by phosphorylating the insulin receptor substrate-1 (IRS-1) on serine residues [53]. The phosphatase Dusp-9 is a protein that inhibits the activity of JNK [42]. Our results show that CR increases the expression of Dusp-9 (Table 1, Fig 8). Thus, high levels of Dusp-9 may increase insulin sensitivity by inhibiting factors such as JNK. In fact, CR mice are extremely insulin sensitive as indicated by the rapid clearance of exogenous glucose in the GTT assay. Moreover, injection of exogenous insulin to CR mice leads to severe hypoglycemia which can result in death. For this reason, CR mice were not used in the ITT studies. In contrast, decreased Dusp-9 can lead to insulin resistance. In effect, Dusp-9 levels were decreased in insulin-resistant obese mice consuming the HF diet. Further studies on the phosphorylation of JNK and insulin pathway signaling factors will provide better insight into alcohol- and CR-induced insulin sensitivity and HF-associated insulin resistance.

On the other hand, phosphatases that impair the insulin pathway may be elevated in obesity. In fact, obese human adipose tissue has increased protein-tyrosine phosphatase (PTPase) activity, which can dephosphorylate and inactivate the insulin receptor kinase, leading to inhibition of IRS-1 phosphorylation [54]. In agreement with this, we found that the mRNA of Ptpra was elevated in adipose tissue of obese mice compared to Control mice (Table 2). As expected, the levels of Ptpra were decreased in CR mice. This suggests that calorie restriction and obesity may affect insulin sensitivity by affecting the balance of phosphatases such as Dusp-9 and Ptpra. Alcohol increased Dusp-9 mRNA levels but had no obvious effect on Ptpra, indicating yet once more that alcohol may improve insulin sensitivity through overlapping but distinct mechanisms compared to body weight.

## Conclusion

In summary, our studies provide a unique experimental design that can be used to better understand how body fat (lean vs. obese) and alcohol consumption affect insulin resistance. We show that obesity induced insulin resistance and that both calorie restriction and alcohol consumption promoted insulin sensitivity. Because adipose tissue is associated with the development of insulin resistance and type-2 diabetes, we isolated total RNA from WAT, selected genes involved in inflammation and energy metabolism, as well as insulin and growth signaling pathways, to evaluate the effect of diet and alcohol on their expression. Results indicate that HF induced Il-6, Il-1α, and Cd68 pro-inflammatory factors in WAT, while CR had the opposite effect on Il-6 and Il-1α expression. Alcohol oppressed different inflammatory factors including Retnlb and Mif. CR and alcohol increased the anti-inflammatory factors Il-10 and Adrbk1. Genes involved in energy homeostasis and insulin signaling such as Ptgds, Lpin2, Lpin3, and Dusp9 were significantly affected by both diet and alcohol; while, Angptl6, Kcnj11, and Ptpra were affected by diet only. Well established metabolic factors such as Gck, Ins1, Pltp, Apob, and Pck1 were also down-regulated by HF and up-regulated by CR and alcohol (Table 1). This work not only indicates that alcohol and diet have overlapping but distinct pathways in regulating insulin sensitivity, but also provides a foundation for studying target pathways that are involved in alcohol- and diet-regulated insulin sensitivity.

## Competing interests

The authors declare that they have no competing interests.

## Authors' contributions

QXP measured gene expression profiles by StellARray and qPCR and helped draft the manuscript. JH carried out the animal study and collected tissues for gene expression analyses. VBH participated in the design and helped draft the manuscript. NPN conceived the study and participated in its design and coordination. All authors read and approved the final manuscript.

## References

[B1] Must A Spadano J Coakley EH Field AE Colditz G Dietz WH The disease burden associated with overweight and obesity JAMA 1999 282 16 1523 9 10.1001/jama.282.16.1523 10546691

[B2] Hu FB Manson JE Stampfer MJ Colditz G Liu S Solomon CG Willett WC Diet, lifestyle, and the risk of type 2 diabetes mellitus in women N Engl J Med 2001 345 11 790 7 10.1056/NEJMoa010492 11556298

[B3] Field AE Coakley EH Must A Spadano JL Laird N Dietz WH Rimm E Colditz GA Impact of overweight on the risk of developing common chronic diseases during a 10-year period Arch Intern Med 2001 161 13 1581 6 10.1001/archinte.161.13.1581 11434789

[B4] Urek R Crncevic-Urek M Cubrilo-Turek M Obesity--a global public health problem Acta Med Croatica 2007 61 2 161 4 17585471

[B5] McMillan DC Sattar N McArdle CS ABC of obesity. Obesity and cancer BMJ 2006 333 7578 1109 11 10.1136/bmj.39042.565035.BE1 17124223 PMC1661751

[B6] Cooper C Inskip H Croft P Campbell L Smith G McLaren M Coggon D Individual risk factors for hip osteoarthritis: obesity, hip injury, and physical activity Am J Epidemiol 1998 147 6 516 22 9521177 10.1093/oxfordjournals.aje.a009482

[B7] Bray GA Health hazards of obesity Endocrinol Metab Clin North Am 1996 25 4 907 19 10.1016/S0889-8529(05)70361-3 8977052

[B8] Koppes LL Dekker JM Hendriks HF Bouter LM Heine RJ Moderate alcohol consumption lowers the risk of type 2 diabetes: a meta-analysis of prospective observational studies Diabetes Care 2005 28 3 719 25 10.2337/diacare.28.3.719 15735217

[B9] Davies MJ Baer DJ Judd JT Brown ED Campbell WS Taylor PR Effects of moderate alcohol intake on fasting insulin and glucose concentrations and insulin sensitivity in postmenopausal women: a randomized controlled trial JAMA 2002 287 19 2559 62 10.1001/jama.287.19.2559 12020337

[B10] Calabro P Limongelli G Pacileo G Di Salvo G Golino P Calabro R The role of adiposity as a determinant of an inflammatory milieu J Cardiovasc Med (Hagerstown) 9 5 450 60 18403996 10.2459/JCM.0b013e3282eee9a8

[B11] Petersen KF Shulman GI Etiology of insulin resistance Am J Med 2006 119 5 Suppl 1 S10 6 10.1016/j.amjmed.2006.01.009 16563942 PMC2995525

[B12] Joosten MM Beulens JW Kersten S Hendriks HF Moderate alcohol consumption increases insulin sensitivity and ADIPOQ expression in postmenopausal women: a randomised, crossover trial Diabetologia 2008 51 8 1375 81 10.1007/s00125-008-1031-y 18504547 PMC2491412

[B13] Bluher S Ziotopoulou M Bullen JW Jr Moschos SJ Ungsunan L Kokkotou E Maratos-Flier E Mantzoros CS Responsiveness to peripherally administered melanocortins in lean and obese mice Diabetes 2004 53 1 82 90 10.2337/diabetes.53.1.82 14693701

[B14] Weisberg SP McCann D Desai M Rosenbaum M Leibel RL Ferrante AW Jr Obesity is associated with macrophage accumulation in adipose tissue J Clin Invest 2003 112 12 1796 808 14679176 10.1172/JCI19246 PMC296995

[B15] Bouloumie A Curat CA Sengenes C Lolmede K Miranville A Busse R Role of macrophage tissue infiltration in metabolic diseases Curr Opin Clin Nutr Metab Care 2005 8 4 347 54 10.1097/01.mco.0000172571.41149.52 15930956

[B16] Xu H Barnes GT Yang Q Tan G Yang D Chou CJ Sole J Nichols A Ross JS Tartaglia LA Chen H Chronic inflammation in fat plays a crucial role in the development of obesity-related insulin resistance J Clin Invest 2003 112 12 1821 30 14679177 10.1172/JCI19451 PMC296998

[B17] Surmi BK Hasty AH Macrophage infiltration into adipose tissue: initiation, propagation and remodeling Future Lipidol 2008 3 5 545 556 10.2217/17460875.3.5.545 18978945 PMC2575346

[B18] Malhi H Gores GJ Molecular mechanisms of lipotoxicity in nonalcoholic fatty liver disease Semin Liver Dis 2008 28 4 360 9 10.1055/s-0028-1091980 18956292 PMC2908270

[B19] Guichard C Moreau R Pessayre D Epperson TK Krause KH NOX family NADPH oxidases in liver and in pancreatic islets: a role in the metabolic syndrome and diabetes? Biochem Soc Trans 2008 36 Pt 5 920 9 10.1042/BST0360920 18793162

[B20] Wojtczak L Schonfeld P Effect of fatty acids on energy coupling processes in mitochondria Biochim Biophys Acta 1993 1183 1 41 57 10.1016/0005-2728(93)90004-Y 8399375

[B21] Eizirik DL Cardozo AK Cnop M The role for endoplasmic reticulum stress in diabetes mellitus Endocr Rev 2008 29 1 42 61 10.1210/er.2007-0015 18048764

[B22] Nunez NP Perkins SN Smith NC Berrigan D Berendes DM Varticovski L Barrett JC Hursting SD Obesity accelerates mouse mammary tumor growth in the absence of ovarian hormones Nutr Cancer 2008 60 4 534 41 10.1080/01635580801966195 18584488

[B23] Akilesh S Shaffer DJ Roopenian D Customized molecular phenotyping by quantitative gene expression and pattern recognition analysis Genome Res 2003 13 7 1719 27 10.1101/gr.533003 12840047 PMC403745

[B24] Rozen S Skaletsky H Primer3 on the WWW for general users and for biologist programmers Methods Mol Biol 2000 132 365 86 10547847 10.1385/1-59259-192-2:365

[B25] Hong J Smith RR Harvey AE Nunez NP Alcohol consumption promotes insulin sensitivity without affecting body fat levels Int J Obes (Lond) 2009 33 2 197 203 10.1038/ijo.2008.266 19125162

[B26] Enriori PJ Evans AE Sinnayah P Cowley MA Leptin resistance and obesity Obesity (Silver Spring) 2006 14 Suppl 5 254S 258S 10.1038/oby.2006.319 17021377

[B27] Mohapatra J Sharma M Singh S Pandya G Chatterjee A Balaraman R Patel PR Jain MR Involvement of adipokines in rimonabant-mediated insulin sensitivity in ob/ob mice J Pharm Pharmacol 2009 61 11 1493 8 10.1211/jpp/61.11.0008 19903374

[B28] Mantzoros CS Li T Manson JE Meigs JB Hu FB Circulating adiponectin levels are associated with better glycemic control, more favorable lipid profile, reduced inflammation in women with type 2 diabetes J Clin Endocrinol Metab 2005 90 8 4542 8 10.1210/jc.2005-0372 15914524

[B29] de Luca C Olefsky JM Inflammation and insulin resistance FEBS Lett 2008 582 1 97 105 10.1016/j.febslet.2007.11.057 18053812 PMC2246086

[B30] Wang P Mariman E Renes J Keijer J The secretory function of adipocytes in the physiology of white adipose tissue J Cell Physiol 2008 216 1 3 13 10.1002/jcp.21386 18264975

[B31] Shoelson SE Herrero L Naaz A Obesity, inflammation, and insulin resistance Gastroenterology 2007 132 6 2169 80 10.1053/j.gastro.2007.03.059 17498510

[B32] Netea MG Joosten LA Lewis E Jensen DR Voshol PJ Kullberg BJ Tack CJ van Krieken H Kim SH Stalenhoef AF Loo FA van de Verschueren I Pulawa L Akira S Eckel RH Dinarello CA Berg W van den Meer JW van der Deficiency of interleukin-18 in mice leads to hyperphagia, obesity and insulin resistance Nat Med 2006 12 6 650 6 10.1038/nm1415 16732281

[B33] Nagata A Suzuki Y Igarashi M Eguchi N Toh H Urade Y Hayaishi O Human brain prostaglandin D synthase has been evolutionarily differentiated from lipophilic-ligand carrier proteins Proc Natl Acad Sci USA 1991 88 9 4020 4 10.1073/pnas.88.9.4020 1902577 PMC51585

[B34] Ragolia L Palaia T Hall CE Maesaka JK Eguchi N Urade Y Accelerated glucose intolerance, nephropathy, and atherosclerosis in prostaglandin D2 synthase knock-out mice J Biol Chem 2005 280 33 29946 55 10.1074/jbc.M502927200 15970590

[B35] Ashcroft FM Harrison DE Ashcroft SJ Glucose induces closure of single potassium channels in isolated rat pancreatic beta-cells Nature 1984 312 5993 446 8 10.1038/312446a0 6095103

[B36] Gloyn AL Weedon MN Owen KR Turner MJ Knight BA Hitman G Walker M Levy JC Sampson M Halford S McCarthy MI Hattersley AT Frayling TM Large-scale association studies of variants in genes encoding the pancreatic beta-cell KATP channel subunits Kir6.2 (KCNJ11) and SUR1 (ABCC8) confirm that the KCNJ11 E23K variant is associated with type 2 diabetes Diabetes 2003 52 2 568 72 10.2337/diabetes.52.2.568 12540637

[B37] Gloyn AL Pearson ER Antcliff JF Proks P Bruining GJ Slingerland AS Howard N Srinivasan S Silva JM Molnes J Edghill EL Frayling TM Temple IK Mackay D Shield JP Sumnik Z van Rhijn A Wales JK Clark P Gorman S Aisenberg J Ellard S Njolstad PR Ashcroft FM Hattersley AT Activating mutations in the gene encoding the ATP-sensitive potassium-channel subunit Kir6.2 and permanent neonatal diabetes N Engl J Med 2004 350 18 1838 49 10.1056/NEJMoa032922 15115830

[B38] Sakura H Ammala C Smith PA Gribble FM Ashcroft FM Cloning and functional expression of the cDNA encoding a novel ATP-sensitive potassium channel subunit expressed in pancreatic beta-cells, brain, heart and skeletal muscle FEBS Lett 1995 377 3 338 44 10.1016/0014-5793(95)01369-5 8549751

[B39] Yang Q Graham TE Mody N Preitner F Peroni OD Zabolotny JM Kotani K Quadro L Kahn BB Serum retinol binding protein 4 contributes to insulin resistance in obesity and type 2 diabetes Nature 2005 436 7049 356 62 10.1038/nature03711 16034410

[B40] Oike Y Akao M Kubota Y Suda T Angiopoietin-like proteins: potential new targets for metabolic syndrome therapy Trends Mol Med 2005 11 10 473 9 10.1016/j.molmed.2005.08.002 16154386

[B41] Yao-Borengasser A Varma V Bodles AM Rasouli N Phanavanh B Lee MJ Starks T Kern LM Spencer HJ III Rashidi AA McGehee RE Jr Fried SK Kern PA Retinol binding protein 4 expression in humans: relationship to insulin resistance, inflammation, and response to pioglitazone J Clin Endocrinol Metab 2007 92 7 2590 7 10.1210/jc.2006-0816 17595259 PMC2893415

[B42] Emanuelli B Eberle D Suzuki R Kahn CR Overexpression of the dual-specificity phosphatase MKP-4/DUSP-9 protects against stress-induced insulin resistance Proc Natl Acad Sci USA 2008 105 9 3545 50 10.1073/pnas.0712275105 18296638 PMC2265194

[B43] Huang SM Hancock MK Pitman JL Orth AP Gekakis N Negative regulators of insulin signaling revealed in a genome-wide functional screen PLoS One 2009 4 9 e6871 10.1371/journal.pone.0006871 19727444 PMC2731165

[B44] Campfield LA Smith FJ Guisez Y Devos R Burn P Recombinant mouse OB protein: evidence for a peripheral signal linking adiposity and central neural networks Science 1995 269 5223 546 9 10.1126/science.7624778 7624778

[B45] Trivedi SG Newson J Rajakariar R Jacques TS Hannon R Kanaoka Y Eguchi N Colville-Nash P Gilroy DW Essential role for hematopoietic prostaglandin D2 synthase in the control of delayed type hypersensitivity Proc Natl Acad Sci USA 2006 103 13 5179 84 10.1073/pnas.0507175103 16547141 PMC1458814

[B46] Ferguson PJ Chen S Tayeh MK Ochoa L Leal SM Pelet A Munnich A Lyonnet S Majeed HA El-Shanti H Homozygous mutations in LPIN2 are responsible for the syndrome of chronic recurrent multifocal osteomyelitis and congenital dyserythropoietic anaemia (Majeed syndrome) J Med Genet 2005 42 7 551 7 10.1136/jmg.2005.030759 15994876 PMC1736104

[B47] Oike Y Yasunaga K Ito Y Matsumoto S Maekawa H Morisada T Arai F Nakagata N Takeya M Masuho Y Suda T Angiopoietin-related growth factor (AGF) promotes epidermal proliferation, remodeling, and regeneration Proc Natl Acad Sci USA 2003 100 16 9494 9 10.1073/pnas.1531901100 12871997 PMC170946

[B48] Oike Y Akao M Yasunaga K Yamauchi T Morisada T Ito Y Urano T Kimura Y Kubota Y Maekawa H Miyamoto T Miyata K Matsumoto S Sakai J Nakagata N Takeya M Koseki H Ogawa Y Kadowaki T Suda T Angiopoietin-related growth factor antagonizes obesity and insulin resistance Nat Med 2005 11 4 400 8 10.1038/nm1214 15778720

[B49] Schaeffer P Bernat A Arnone M Manara L Gallas JF Dol-Gleizes F Millet L Grosset A Herbert JM Effect of SR58611A, a potent beta-3 adrenoceptor agonist, on cutaneous wound healing in diabetic and obese mice Eur J Pharmacol 2006 529 1-3 172 8 10.1016/j.ejphar.2005.11.005 16325798

[B50] Overweight, obesity, health risk. National Task Force on the Prevention and Treatment of Obesity Archives of Internal Medicine 2000 160 7 898 904 10.1001/archinte.160.7.898 10761953

[B51] Hirosumi J Tuncman G Chang L Gorgun CZ Uysal KT Maeda K Karin M Hotamisligil GS A central role for JNK in obesity and insulin resistance Nature 2002 420 6913 333 6 10.1038/nature01137 12447443

[B52] Fujishiro M Gotoh Y Katagiri H Sakoda H Ogihara T Anai M Onishi Y Ono H Abe M Shojima N Fukushima Y Kikuchi M Oka Y Asano T Three mitogen-activated protein kinases inhibit insulin signaling by different mechanisms in 3T3-L1 adipocytes Mol Endocrinol 2003 17 3 487 97 10.1210/me.2002-0131 12554784

[B53] Aguirre V Werner ED Giraud J Lee YH Shoelson SE White MF Phosphorylation of Ser307 in insulin receptor substrate-1 blocks interactions with the insulin receptor and inhibits insulin action J Biol Chem 2002 277 2 1531 7 10.1074/jbc.M101521200 11606564

[B54] Mander A Hodgkinson CP Sale GJ Knock-down of LAR protein tyrosine phosphatase induces insulin resistance FEBS Lett 2005 579 14 3024 8 10.1016/j.febslet.2005.04.057 15896785

